# Effect of Job Training and Work Environment on Professionalism Among Direct Long-Term Care Workers

**DOI:** 10.3390/bs15121731

**Published:** 2025-12-15

**Authors:** Chae Yoon Kim, Jeong Mi Lim, Bum Jung Kim

**Affiliations:** 1 Dokyung Older Adult Welfare Center, Suwon 16686, Republic of Korea; 2 Department of Social Welfare, Gyeongsang National University, 501 Jinju-daero, Jinju-si 52828, Republic of Korea; 3 Center for Policy Research on Aging, University of California, 337 Charles E. Young Drive East, Los Angeles, CA 90095, USA

**Keywords:** long-term care workers, professionalism, job training, work environment, workforce development, aging society

## Abstract

This study examined the associations of job training and work environment with professionalism among direct long-term care (LTC) workers in South Korea. Given the cross-sectional design, the findings reflect statistical associations rather than causal relationships. A survey of 264 LTC workers was analyzed using descriptive statistics, correlations, and hierarchical regression. Model fit improved from Model 1 to Model 3 (R^2^ = 0.370), and regression assumptions—including normality, homoscedasticity, and multicollinearity—were verified (all VIFs < 2.5). Work environment factors showed the strongest associations with professionalism. In the fully adjusted model, work promotion was positively associated (β = 0.177, *p* < 0.05), whereas work hindrance was negatively associated (β = −0.201, *p* < 0.01). Among sociodemographic variables, education (β = 0.183, *p* < 0.01) and monthly income (β = 0.113, *p* < 0.05) were significant. Job training showed no direct association with professionalism, likely reflecting limited variability and repetitive training content across institutions. Enhancing work environments—particularly by increasing recognition and reducing work obstacles—may strengthen professionalism among LTC workers. Job training systems may require redesign to improve relevance and effectiveness. Because data were drawn from a single region (Gyeonggi-do) and rely on self-report measures, generalizability is limited. Future studies should include multi-regional or longitudinal designs to deepen the understanding of workforce professionalism in aging societies. Practically, these findings suggest that improving recognition systems, reducing workflow barriers, and modernizing standardized training curricula may help strengthen professionalism among long-term care workers.

## 1. Introduction

Countries across the world are facing rapid demographic aging, declining fertility rates, and increasing dependence on formal long-term care (LTC) systems. These demographic shifts intensify pressures on LTC labor markets, making the recruitment, retention, and professionalization of the direct care workforce an international policy priority (OECD, 2020; National Statistical Office Korea, 2023; Stone & Bryant, 2019). South Korea reflects these global trends in an accelerated form: the proportion of older adults has grown rapidly, and functional limitations associated with chronic conditions have increased the demand for formal caregiving. Since the implementation of the national long-term care insurance system in 2008, direct care workers have become the backbone of Korea’s LTC services.

Despite their essential role, direct LTC workers in Korea continue to face persistent challenges, including low wages, limited career mobility, and a lack of recognition as skilled professionals. Similar concerns have been documented globally, where direct care work remains undervalued, characterized by high turnover, job strain, and limited formal training pathways (Campbell et al., 2023; Dall et al., 2022). These pressures underscore the importance of strengthening professionalism—a construct that encompasses not only technical competence but also ethical commitment, autonomy, and professional identity.

While concepts such as expertise, competence, and professionalism are related, they represent distinct dimensions. *Expertise* refers to accumulated knowledge and specialized skills; *competence* reflects the ability to perform tasks effectively; and *professionalism* encompasses broader attitudes, values, and identity associated with one’s occupation (Hall, 1968; Noordegraaf, 2020). For direct LTC workers, professionalism is shaped not only by individual ability but also by structural supports, organizational conditions, and the perceived legitimacy of their work. However, empirical studies examining how job training and work environments shape professionalism within the LTC sector—particularly in East Asian contexts—remain limited (Shim, 2011; Weaver et al., 2022).

International research suggests that high-quality training improves care quality, job satisfaction, and retention, yet training for LTC workers often remains inconsistent, repetitive, or insufficiently aligned with actual job demands (Fujisawa & Colombo, 2019; M. S. Park, 2009). In Korea, mandatory training requirements exist, but previous studies have shown that content overlap, limited instructor capacity, and lack of practical applicability weaken training effectiveness. This raises the question of whether current training systems meaningfully contribute to strengthening professionalism.

In contrast, work environment factors—such as supervisory support, recognition, organizational culture, and workload—have consistently shown strong relationships with professional identity and job commitment across multiple international settings (Morgan et al., 2021; Weaver et al., 2023). Poor working conditions, inadequate compensation, and work hindrance are known to reduce workers’ sense of autonomy and professional worth, suggesting that environmental factors may exert a stronger influence on professionalism than training alone (D. G. Park, 2021; J. K. Park, 2018).

Despite the importance of these issues, few studies have systematically examined the relative contributions of job training and work environment to professionalism among direct LTC workers in South Korea. The existing literature tends to focus on job satisfaction, turnover intention, or burnout rather than professional identity. Furthermore, cross-national research underscores the need for context-specific analyses, as LTC workforce development varies substantially by country (Ministry of Health and Welfare, 2020).

However, prior research has rarely examined job training and work environment together within a single analytic framework, despite increasing international concern about the professionalization of direct care work. This study addresses this gap by integrating structural (training) and contextual (work environment) predictors and analyzing their relative contributions to professionalism. By doing so, it offers a more critical understanding of why training systems may underperform and why organizational factors remain decisive across countries.

To address this gap, the present study examines how job training and work environment relate to professionalism among direct LTC workers in Gyeonggi-do, a region that hosts a large proportion of Korea’s LTC workforce and is often considered demographically and structurally representative of national patterns. By analyzing job training subdimensions and work environment characteristics within a single analytic framework, this study contributes to a clearer understanding of the structural and organizational factors that shape professionalism in aging societies. The findings aim to inform workforce development strategies and policy reforms geared toward strengthening professionalism in the LTC sector.

## 2. Theoretical Background

### 2.1. Expertise, Competence, and Professionalism

The concepts of expertise, competence, and professionalism are often used interchangeably in long-term care research, yet they represent distinct theoretical dimensions. *Expertise* refers to specialized knowledge gained through education and accumulated experience; *competence* denotes the demonstrated ability to perform job tasks effectively; and *professionalism* encompasses broader values, attitudes, and identity elements—such as autonomy, ethical commitment, and public service orientation—that shape how workers view and enact their roles (Hall, 1968; Noordegraaf, 2020).

Hall’s (1968) conceptualization of professionalism highlights both structural and attitudinal dimensions. Structural aspects include systems of formal education, standardized training mechanisms, professional codes of ethics, and organizational infrastructures. Attitudinal aspects reflect internalized values such as belief in self-regulation, sense of vocational calling, and commitment to public service. This dual framework is particularly relevant to LTC work, where professional identity is shaped not only by skill-based training but also by how workers perceive their status, autonomy, and organizational support.

International research emphasizes that professionalism among direct care workers arises from the interplay between knowledge, job-specific competence, and the environment in which workers operate. For instance, the expansion of competency-based training in the United States and parts of Europe demonstrates that formal education alone does not guarantee professional identity unless accompanied by supportive supervision, recognition systems, and organizational culture (Weaver et al., 2023; Campbell et al., 2023). Thus, examining professionalism requires both structural indicators—such as training systems—and contextual factors—such as workplace conditions.

In the Korean LTC context, where direct care work has historically been viewed as low-skilled, understanding these three constructs separately is essential. This study adopts the following conceptual stance: job training primarily relates to the structural aspect of professionalism by enhancing expertise and competence, whereas work environment factors predominantly influence the attitudinal dimension by shaping autonomy, recognition, and professional values (Bae, 2013; C. Y. Cho, 2007; Y. J. Cho, 2011).

### 2.2. Job Training and Professionalism

Job training is widely recognized as a pathway to establishing and enhancing professionalism, particularly in occupations where formal qualifications and continuing education are essential for service quality. Prior studies have found that high-quality training improves competence, increases job satisfaction, and enhances care quality across multiple LTC settings (Fujisawa & Colombo, 2019). International evidence also shows that standardized and competency-based training frameworks contribute to stronger professional identity among direct care workers.

However, challenges persist. In many countries, including South Korea, LTC training programs remain variable in content, duration, and delivery. Repetitive curricula, inconsistent instructor quality, and limited opportunities for hands-on practice reduce the perceived relevance of training among care workers. Empirical studies in Korea have noted limited training effects when programs do not adequately reflect real caregiving tasks or when workers receive the same content repeatedly without progression or specialization. These structural issues raise questions about whether existing training effectively supports professionalism beyond meeting regulatory requirements.

From a theoretical perspective, job training strengthens professionalism only when it enhances both expertise (knowledge) and competence (practical ability) in ways that workers perceive as meaningful and applicable. When training is insufficiently differentiated, highly standardized, or poorly aligned with job realities, its contribution to professionalism may be limited. This study analyzes whether subdimensions of training—such as training content, instructors, methods, and environment—are associated with professionalism within Korea’s LTC system.

### 2.3. Work Environment and Professionalism

Work environment factors—including supervisory support, recognition, organizational culture, workload, and interpersonal relationships—are strongly associated with professional identity across care sectors globally. Prior research in Europe, North America, and Asia demonstrates that positive work environments enhance workers’ sense of autonomy, ethical responsibility, and organizational commitment, all of which contribute to professionalism (Cox & Heinz, 2021; Morgan et al., 2021; Stone & Bryant, 2019). Supportive leadership, fair compensation systems, and opportunities for participation in decision-making have been identified as key determinants of how care workers view their professional roles.

Conversely, high workloads, time pressure, lack of recognition, and role ambiguity undermine workers’ sense of control and professional worth. These work-hindering factors are linked to decreased job commitment, burnout, turnover intention, and diminished professional identity (Gil, 2015; H. J. Kwon, 2021; Weaver et al., 2023). In Korea, long-term care workers report work interruptions, emotional demands, and insufficient time allocated per client, which intensify stress and limit opportunities to enact care in ways consistent with professional values (Jo, 2015; H. Kim et al., 2021; T. Kim, 2018; S. Lee, 2001).

Drawing from Hall’s (1968) attitudinal dimension of professionalism, work environment factors shape how workers interpret their occupational identity and whether they view their role as skilled and meaningful. This study examines five subdimensions—personnel policy, job environment, organizational characteristics, work promotion, and work hindrance—to assess whether environmental conditions exert stronger influence on professionalism than training alone (Lim, 2021).

Overall, the theoretical framework can be summarized as positioning job training as a structural component of professionalism and the work environment as a contextual and attitudinal component, reflecting how professional identity forms through both skills and workplace conditions.

## 3. Methods

### 3.1. Research Model and Hypotheses

This study examined the associations of job training and work environment with professionalism among direct long-term care (LTC) workers (Table 1).

Based on Hall’s (1968) structural and attitudinal framework of professionalism, job training was conceptualized as a structural factor, whereas work environment was framed as an attitudinal factor influencing professional identity.

Two hypotheses were formulated:

**H1.**  *Job training is positively associated with professionalism.*

**H2.**  *Work environment is positively associated with professionalism.*

### 3.2. Data Collection

#### Study Setting and Sampling

Data were collected between August and September 2021 from LTC agencies in Gyeonggi-do, Republic of Korea. Gyeonggi-do was selected because it contains the largest proportion of LTC institutions nationwide and is widely considered demographically and structurally representative of national LTC patterns. However, the use of a single region limits external validity. A convenience sampling strategy was used due to restrictions related to COVID-19 and institutional access. To recruit participants, LTC centers were contacted by phone and email, informed of the study purpose, and invited to participate. Centers that agreed to participate distributed printed questionnaires to their direct LTC workers. A total of 269 questionnaires were distributed, and 264 were returned with valid responses (response rate = 98.1%). Although the LTC workforce is predominantly female in Korea, the gender composition of this sample (98% women) remains a potential source of sampling bias. The study was approved by the Institutional Review Board of Chung-Ang University (IRB No. 1041078–202104-HRSB–111–01). All participants provided informed consent.

### 3.3. Measurement Tools and Data Analysis Methods

#### 3.3.1. Professionalism (Dependent Variable)

Professionalism was measured using a validated Korean adaptation of Hall’s (1968) professionalism scale, originally operationalized by Snizek (1972) and later modified for LTC workers by J. Lee (2015). The instrument consists of five subdomains (proactivity, regulation beliefs, autonomy, public service beliefs, calling), totaling 25 items. Higher scores indicate higher professionalism. Internal consistency in this study was acceptable (Cronbach’s α = 0.833). Construct validity has been established in prior Korean LTC studies using this scale. A confirmatory factor analysis (CFA) was not performed in the present study because the instrument has been repeatedly validated in previous research, and the primary objective was to examine associations rather than evaluate measurement structure.

#### 3.3.2. Job Training (Independent Variable)

Job training was assessed across five subdimensions: training content, training method, instructor quality, training environment, and motivation for participation (28 items). These subdimensions were adapted from validated instruments used in studies by Yoon (2008), Won (2008), J. Y. Park (2012), H. Y. Kwon (2012), Kwak (2013), and C. Lee (2015).

Internal consistency for all subdomains exceeded the acceptable threshold (Cronbach’s α = 0.859–0.918). Prior research has supported the construct validity of these subdomains in Korean LTC settings. While a CFA was not performed, reliability and prior validation provide sufficient justification for the use of this instrument.

#### 3.3.3. Work Environment (Independent Variable)

Work environment was measured using subscales adapted from Amabile and Conti’s (1999) creativity-supportive work environment scale, the Job Descriptive Index (Smith et al., 1969), and Korean LTC studies (Shin, 2010; H. Lee, 2011; J. U. Park, 2014). The five subdomains included personnel policy, job environment, organizational characteristics, work promotion, and work hindrance (18 items). Internal consistency ranged from 0.696 to 0.890.

### 3.4. Data Analysis

Data analysis was conducted using SPSS 28. First, descriptive statistics were used to summarize the sociodemographic characteristics of participants. Second, internal consistency for each subscale related to job training, work environment, and professionalism was examined using Cronbach’s α. Third, Pearson correlation analyses were performed to identify the relationships among key study variables and to preliminarily assess multicollinearity. Finally, hierarchical regression analysis was conducted to examine the associations between job training, work environment, and professionalism.

Hierarchical regression was selected because it allows for the sequential entry of predictor groups, enabling an assessment of the incremental explanatory power of each set of variables. In Model 1, sociodemographic characteristics were entered to establish a baseline. In Model 2, job training subdimensions were added to examine their contribution beyond personal characteristics. In Model 3, work environment variables were introduced to evaluate their additional explanatory value. This approach is consistent with Hall’s (1968) framework, which conceptualizes professionalism as shaped by both structural factors (such as training) and attitudinal factors (such as work environment).

Prior to conducting regression analyses, major analytical assumptions were verified. The normality of residuals was examined using Q–Q plots, and homoscedasticity was assessed through visual inspection of residual scatterplots. Multicollinearity was evaluated using variance inflation factors (VIFs), all of which were below 2.5, indicating no multicollinearity concerns. Although the data were collected through convenience sampling—limiting representativeness—the analytical approach remains appropriate for examining statistical associations rather than population-level inferences.

A sensitivity analysis, such as bootstrapping, was considered but was not feasible due to the structure of the dataset. To enhance the robustness of estimates, however, regression analyses were conducted using robust standard errors. A priori power analysis was not conducted due to institutional and logistical constraints, and this should be considered when interpreting the findings.

Although no a priori power analysis was conducted, the sample size of 264 exceeds commonly recommended minimum thresholds for multiple regression (e.g., N > 15 × number of predictors), suggesting adequate statistical power for the present analysis. Reviewer suggestions regarding exploratory factor analysis (EFA) are appreciated; however, EFA was not conducted because the study did not aim to validate measurement structure but rather to examine associations using instruments already validated in prior Korean LTC studies. Nonetheless, we acknowledge that several subdimensions, particularly within the work environment scale, include only three or four items, which may limit construct validity and should be considered a measurement limitation.

## 4. Results

### 4.1. Sociodemographic Characteristics

Among the 264 participants, the majority were women (98.1%), reflecting the gendered composition of Korea’s LTC workforce. The average age was 60.9 years, and most participants were married and had at least a high school education. Monthly income averaged approximately 900 USD, and caregiving experience varied, with about one-third having more than 60 months of experience. Detailed characteristics are presented in Table 2.

### 4.2. Reliability and Correlation Analysis

All study variables demonstrated acceptable internal consistency, with Cronbach’s α values exceeding 0.69 for all subscales (Table 3). Correlation analyses indicated significant positive associations between work environment subdimensions—such as personnel policy, job environment, organizational characteristics, and work promotion—and professionalism (Table 4). Work hindrance was negatively correlated with professionalism. Job training subdimensions were positively correlated with professionalism but at lower magnitudes than work environment factors. No multicollinearity issues were identified in the correlation matrix.

### 4.3. Hierarchical Regression Analysis

Hierarchical regression analysis was conducted to examine the associations of job training and work environment with professionalism. Prior to running the models, regression assumptions were assessed, and all were found to be satisfactory; in particular, variance inflation factors (VIFs) were below 2.5 for all predictors, indicating no multicollinearity concerns (Table 5).

The first model included only sociodemographic characteristics and was not statistically significant as a whole. Nevertheless, education level and monthly income showed early indications of positive relationships with professionalism, although these associations did not reach significance in this initial step. When job training subdimensions were added in Model 2, the explained variance increased substantially (R^2^ = 0.246). Despite this increase, none of the job training components—training content, method, training environment, instructor quality, or participation motivation—were significantly associated with professionalism after controlling for sociodemographic variables. This pattern likely reflects the limited variability and high degree of standardization in mandated LTC training programs, which may constrain their ability to distinguish differences in professionalism among workers.

Model 3 incorporated work environment variables and yielded the highest explanatory power (R^2^ = 0.370). Several predictors emerged as significant. Education maintained a positive relationship with professionalism (β = 0.183, *p* < 0.01), and monthly income also showed a significant positive association (β = 0.113, *p* < 0.05). Among the work environment factors, work promotion demonstrated a meaningful positive association with professionalism (β = 0.177, *p* < 0.05), suggesting that recognition, encouragement, and supportive feedback contribute to a stronger sense of professional identity. In contrast, work hindrance exhibited the strongest effect in the model, showing a significant negative association with professionalism (β = −0.201, *p* < 0.01). Workers who experienced time pressure, frequent interruptions, or rigid procedures tended to report lower levels of professionalism. Other work environment subdimensions—such as personnel policy, job environment, organizational characteristics, and work facilitation—were positively correlated with professionalism at the bivariate level but did not reach statistical significance in the fully adjusted model.

For additional robustness, 95% confidence intervals were examined for the significant predictors, and all intervals excluded zero, reinforcing the stability of the observed associations. Overall, the findings indicate that while job training does not significantly account for variations in professionalism, work environment factors—particularly work promotion and work hindrance—play a more substantial role in shaping the professional identity of direct LTC workers.

## 5. Discussion

### 5.1. Interpretation of Key Findings

This study examined how job training and work environment relate to professionalism among direct long-term care (LTC) workers in South Korea. The findings demonstrate that work environment factors—particularly work promotion and work hindrance—were more strongly associated with professionalism than job training. These results align with Hall’s (1968) framework, which suggests that professionalism is shaped not only by structural elements such as training but also by attitudinal elements formed through organizational context and daily work experiences.

The strong negative association between work hindrance and professionalism indicates that time pressure, frequent interruptions, and rigid procedures may erode workers’ sense of autonomy and professional identity. Conversely, work promotion—characterized by recognition of effort, encouragement, and supportive feedback—was positively associated with professionalism. These patterns suggest that LTC workers build their professional identity not merely through formal training but through interpersonal and organizational experiences that affirm their role and expertise.

Job training, however, was not significantly associated with professionalism after controlling for sociodemographic and environmental factors. This nonsignificance may reflect the highly standardized and repetitive nature of Korea’s mandated LTC training programs, which limits variability across workers. When training curriculum, content, and instructor quality are largely uniform across institutions and when required hours are minimal, training may have limited ability to differentiate professionalism levels. Additionally, workers often report repeated exposure to the same training topics, reducing perceived relevance and diminishing training effects.

The significance of education and income further suggests that professionalism may be linked to broader structural indicators of social status, skill development, and perceived value in the workplace. Workers with higher educational attainment or greater income may perceive themselves as more skilled or valued, contributing to a stronger professional identity.

### 5.2. Comparison with International Research

The findings are consistent with international literature emphasizing that work environment factors—rather than training alone—play a decisive role in shaping professional identity among care workers. Studies from the United States and Europe have shown that supportive supervision, recognition systems, and opportunities for meaningful decision-making strongly influence professional values, autonomy, and job commitment among direct care staff (Weaver et al., 2023; Campbell et al., 2023). Similarly, research in Japan and the United Kingdom has documented that work hindrance, such as excessive workload and time constraints, contributes to reduced professional pride and greater turnover intention.

In comparison, Korea’s LTC training system remains more standardized and less differentiated than systems in countries such as Germany or the Netherlands, where tiered certification pathways and competency-based progression contribute to stronger professionalization. The limited variation in Korean training programs may explain why job training showed no direct association with professionalism in this study, whereas international studies have found stronger effects when training content is specialized, practical, and linked to career advancement.

Furthermore, prior research from Asian and Western countries emphasizes the importance of organizational culture and leadership in fostering professionalism in care settings. The present findings reinforce this pattern, suggesting that improvements in workplace recognition and reductions in work hindrance may yield greater improvements in professionalism than adjustments to training alone.

These findings contrast with studies in countries such as Germany, the Netherlands, and parts of the United States where specialized or tiered training systems have demonstrated meaningful improvements in competence, job satisfaction, and professional identity. The discrepancy may reflect Korea’s highly standardized and repetitive training structure, which offers limited differentiation or progression. Thus, training effects in this study should be interpreted within Korea’s unique institutional context, where training may serve more as regulatory compliance than as professional development.

### 5.3. Implications for Policy and Practice

The study offers several implications for efforts to strengthen the LTC workforce. First, organizational strategies that reduce work interruptions, clarify workflow expectations, and address time constraints may help enhance professionalism. Ensuring adequate staffing levels, improving task allocation systems, and minimizing procedural rigidity could reduce work hindrance and increase workers’ ability to deliver care aligned with professional values.

Second, recognition-based systems, such as supervisor feedback, acknowledgment of effort, and opportunities for idea sharing, appear to play a meaningful role in shaping workers’ professional identity. Institutions may consider implementing structured recognition programs, peer-support systems, or leadership training that equip supervisors to provide encouragement and constructive feedback.

Third, the nonsignificant association between job training and professionalism suggests a need to redesign Korea’s LTC training system. Training programs may benefit from incorporating practical, scenario-based modules, expanding instructor competency evaluations, and developing tiered education pathways that allow workers to progress professionally. Updating repetitive course materials and providing differentiated training based on experience levels could also increase training effectiveness.

Finally, income effects underscore the continued importance of compensation reform. Fair wage structures that reflect experience, skill level, and performance may enhance workers’ perception of professional value and help retain skilled caregivers in the sector.

Taken together, the findings suggest that workplace conditions play a more decisive role in shaping LTC workers’ professionalism than training alone, and that discussions of workforce professionalization must account for both structural and contextual factors.

### 5.4. Limitations and Future Directions

Several limitations should be acknowledged. First, the study used a cross-sectional design, preventing causal inferences; all associations should therefore be interpreted as correlational. Second, data were collected from a single region (Gyeonggi-do), limiting generalizability to the broader national LTC workforce. Third, the convenience sampling method and the predominance of female participants (98%) may introduce sampling bias. Fourth, self-report measures are subject to social desirability and recall bias, and workers may have experienced survey fatigue due to the length and repetitiveness of training-related items. Fifth, job training subdimensions showed limited variability, which may partially explain their nonsignificance.

Future research could employ longitudinal designs to examine changes in professionalism over time or mixed-method approaches to explore workers’ perceptions of professionalism more deeply. Comparative studies across regions or countries may also help identify structural and cultural factors that support professional identity in LTC settings. Additionally, future studies should consider incorporating variables such as job autonomy, supervisory quality, and emotional labor to further illuminate the determinants of professionalism.

## 6. Conclusions

This study examined how job training and work environment relate to professionalism among direct long-term care (LTC) workers in South Korea. The findings highlight that professionalism is shaped more strongly by workplace conditions—particularly recognition and work hindrance—than by mandated job training. While job training is an important structural component of Korea’s LTC system, its limited variability and standardized curriculum may constrain its influence on workers’ professional identity. In contrast, environmental factors that affirm workers’ contributions or impede their daily tasks show clear associations with professionalism.

The study offers several contributions. Theoretically, it expands Hall’s (1968) framework by demonstrating how attitudinal and organizational elements shape professionalism within a frontline LTC context. Empirically, it provides one of the few quantitative analyses examining professionalism among direct care workers in Korea, integrating both training- and environment-based predictors within a single analytic model. Methodologically, it illustrates the value of assessing subdimensions of training and work environment rather than relying on aggregated indices.

The findings also underscore practical considerations for strengthening the LTC workforce. Improving recognition systems, reducing workflow barriers, and updating training curricula may help foster stronger professional identity among care workers. Given the rapid aging of Korea’s population and persistent workforce shortages, efforts to enhance professionalism may contribute to improved care quality and retention.

Despite its contributions, the study is limited by its cross-sectional design, regional focus, and reliance on self-reported measures. Future research should incorporate longitudinal or multi-regional data and explore additional factors—such as supervisory quality, job autonomy, and emotional labor—that may further explain variations in professionalism.

Overall, this study provides insight into the structural and environmental conditions that support professionalism in LTC settings and highlights the importance of organizational efforts that recognize and empower direct care workers.

Ultimately, the findings reinforce an urgent need to further professionalize the LTC sector through improved organizational conditions, differentiated training pathways, and stronger recognition of the specialized skills required in caregiving.

## Figures and Tables

**Table 1:** Research Model.

Independent Variables		Dependent Variable
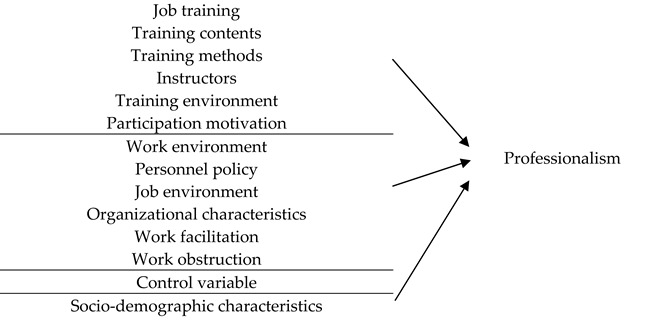		

**Table 2:** Sample Characteristics (N = 264).

Variable	N	%
Age *M (SD)*	60.9 (7.25)	
Gender Male Female	5 259	1.90 98.1
Marital status Single (unmarried/widowed/divorced) Married	7 256	2.70 97.3
Education Elementary Middle High College Graduate	15 67 148 33 1	5.70 25.4 56.1 12.5 0.4
Monthly income (dollars) *M (SD)*	900 (45.61)	
Care work experience Less than 30 months 30 months to less than 60 months 60 months or more	76 89 99	28.8 33.7 37.5
Institution experience Less than 20 months 20 months to less than 40 months 40 months or more	85 91 88	32.2 34.5 33.3
Number of workers Less than 50 50–99 100 or more	132 117 15	50.0 44.3 5.70
Job training Contents Method Environment Instructor Participation motivation	26.33 24.88 21.05 31.02 20.47	3.60 4.16 3.54 4.17 3.70
Work environment Personnel policy Job environment Organizational characteristics Work facilitation Work obstruction	11.93 16.66 16.70 17.46 17.62	2.95 2.96 2.67 4.76 4.65
Professionalism *M (SD)*	94.13 (12.06)	

**Table 3:** Reliability (N = 264).

Category	Item		# of Items	N	Reliability
Independent	Job training	Contents	6	264	0.905
		Method	6	263	0.899
		Environment	5	264	0.859
		Instructor	7	264	0.918
		Participation motivation	5	264	0.861
	Work environment	Personnel policy	3	264	0.696
		Job environment	4	264	0.890
		Organizational characteristics	4	264	0.828
		Work facilitation	5	264	0.899
		Work obstruction	5	264	0.848
Dependent	Professionalism		25	264	0.833

**Table 4:** Correlations among Study Variables for Care Workers (N = 264).

	1	2	3	4	5	6	7	8	9	10	11	12	13	14	15	16	17	18
1. Age	1																	
2. Gender	0.048	1																
3. Marital status	0.496 ***	0.079	1															
4. Education	0.067	−0.280 ***	0.074	1														
5. Income	−0.065	−0.044	−0.130 *	0.074	1													
6. Caregiving experience	0.084	0.134 *	0.076	−0.053	0.064	1												
7. Institution experience	0.036	0.074	0.002	0.029	0.100	0.530 ***	1											
8. # of care workers	0.082	0.053	−0.044	−0.021	0.005	0.009	0.057	1										
9. Training contents	−0.088	−0.043	−0.104	−0.174 **	−0.012	−0.153 *	−0.079	0.210 **	1									
10. Method	−0.085	0.066	−0.068	−0.246 ***	−0.058	−0.082	−0.075	0.172 **	0.722 ***	1								
11. Environment	−0.073	0.032	−0.102	−0.192 **	0.002	−0.095	−0.109	0.176 **	0.709 ***	0.771 ***	1							
12. Instructor	−0.101	0.075	−0.039	−0.210 **	−0.005	0.034	0.050	0.177 **	0.540 ***	0.663 ***	0.608 ***	1						
13. Participation motivation	−0.127 *	0.073	−0.0140 *	−0.187 **	−0.107	−0.077	−0.070	0.139 *	0.557 ***	0.606 ***	0.627 ***	0.556 ***	1					
14. Personnel policy	−0.087	0.099	−0.016	−0.169 **	−0.003	−0.022	−0.043	0.103	0.436 ***	0.472 ***	0.485 ***	0.507 ***	0.500 ***	1				
15. Work environment	−0.092	0.067	−0.057	−0.173 **	0.077	0.036	0.029	0.139 *	0.394 ***	0.448 ***	0.437 ***	0.509 ***	0.407 ***	0.627 ***	1			
16. Organizational characteristics	−0.152 *	0.077	−0.108	−0.186 **	0.008	0.007	0.031	0.155 *	0.486 ***	0.543 ***	0.524 ***	0.550 ***	0.454 ***	0.633 ***	0.710 ***	1		
17. Work facilitation	−0.058	0.110	−0.010	−0.138 *	0.003	0.077	0.079	0.199 **	0.334 ***	0.430 ***	0.380 ***	0.457 ***	0.354 ***	0.580 ***	0.574 ***	0.645 ***	1	
18. Work obstruction	−0.048	−0.099	−0.014	0.076	0.161 **	0.042	0.150 *	0.109	0.072	0.026	0.083	0.081	−0.049	−0.013	0.203 **	0.158 *	−0.083	1
19. Professionalism	−0.119	−0.010	−0.077	0.052	0.095	0.001	0.082	0.126 *	0.350 ***	0.388 ***	0.336 ***	0.401 ***	0.344 ***	0.426 ***	0.384 ***	0.439 ***	0.470 ***	−0.113

* *p* < 0.05, ** *p* < 0.01, *** *p* < 0.001.

**Table 5:** Standardized Coefficients from Robust Hierarchical Regression on Professionalism.

Category		Model 1		Model 2		Model 3	
		B(β)	SE	B(β)	SE	B(β)	SE
Age		0.004 (0.002)	0.107	0.005 (0.003)	0.097	−0.056 (−0.034)	0.090
Gender		−11.194 (−0.128)	6.225	−6.448 (−0.074)	5.641	−5.517 (−0.063)	5.243
Marital status		0.124 (0.002)	5.325	0.410 (0.006)	4.833	−0.096 (−0.001)	4.495
Education		0.809 (0.051)	1.023	2.606 (0.164) **	0.948	2.904 (0.183) **	0.878
Monthly income		0.022 (0.084)	0.016	0.027 (0.104)	0.015	0.029 (0.113) *	0.014
Caregiving experience		−0.479 (−0.033)	1.079	−0.078 (−0.005)	0.982	−0.424 (−0.029)	0.909
Institution experience		1.138 (0.077)	1.077	1.158 (0.078)	0.978	1.471 (0.100)	0.915
# of care workers		2.534 (0.128) *	1.231	0.610 (0.031)	1.137	0.512 (0.026)	1.068
Job training	Content	-	-	0.298 (0.090)	0.290	0.256 (0.077)	0.270
	Method	-	-	0.521 (0.181)	0.292	0.317 (0.110)	0.272
	Environment	-	-	−0.130 (−0.045)	0.278	−0.141 (−0.049)	0.260
	Instructor	-	-	0.687 (0.203) *	0.266	0.356 (0.105)	0.256
	Participation motivation	-	-	0.419 (0.129)	0.247	0.149 (0.046)	0.235
Work environment	Personnel policy	-	-	-	-	0.488 (0.097)	0.383
	Work environment	-	-	-	-	0.238 (0.058)	0.325
	Organizational characteristics	-	-	-	-	0.532 (0.118)	0.392
	Work facilitation	-	-	-	-	0.446 (0.177) *	0.188
	Work obstruction	-	-	-	-	−0.517 (−0.201) **	0.148
Constants		94.882 ***	9.471	46.725 ***	10.966	50.869 ***	10.442
F		1.570		6.237 ***		7.950 ***	
R^2^ (Adj. R^2^)		0.047 (0.017)		0.246 (0.206)		0.370 (0.323)	
ΔR^2^		-		0.199		0.124	

* *p* < 0.05, ** *p* < 0.01, *** *p* < 0.001.

## Data Availability

Data supporting the findings of this study are available from the corresponding author upon reasonable request.
